# Recurring Paralysis and a Race Against Time: A Case of Cauda Equina Syndrome With Delayed Diagnosis and Incomplete Neurological Recovery

**DOI:** 10.7759/cureus.93780

**Published:** 2025-10-03

**Authors:** Pallavi Priya, Praveen Thiruneelakantan, Amani Mokbel, Abuamar Zaidan, Subham Baid

**Affiliations:** 1 Medical Education, Southend University Hospital, Mid and South Essex NHS Foundation Trust, Southend-on-Sea, GBR; 2 Orthopaedics and Trauma, Southend University Hospital, Mid and South Essex NHS Foundation Trust, Southend-on-Sea, GBR; 3 Emergency Medicine, Southend University Hospital, Mid and South Essex NHS Foundation Trust, Southend-on-Sea, GBR; 4 Medicine, Southend University Hospital, Mid and South Essex NHS Foundation Trust, Southend-on-Sea, GBR

**Keywords:** cauda equina syndrome (ces), chronic sciatica, lumbar disc herniation surgery, neurological deficiency, spinal decompression

## Abstract

Cauda equina syndrome (CES) is a rare but critical neurological condition caused by compression of the cauda equina, a collection of lumbosacral nerve roots responsible for lower limb function along with bowel and bladder control. While CES often presents as a sudden and severe onset of symptoms, it can also develop insidiously, mimicking other conditions in its early stages, such as lumbar radiculopathy, spinal stenosis, or urinary tract disorders, often contributing to delayed recognition. Given the variability in clinical presentation, early recognition of suggestive signs and symptoms is crucial to prevent permanent neurological deficits. Delayed recognition may result in irreversible disability despite surgical intervention. We report the case of a 35-year-old male patient with a history of chronic right-sided sciatica, who reported to the emergency department with progressively worsening lower back pain radiating to the right leg, accompanied by bilateral limb weakness and evolving neurological deficits. Urgent MRI imaging revealed a significant L4-L5 disc herniation compressing the cauda equina, prompting emergency surgical decompression. Although the patient initially improved following spinal decompression, he later re-presented with recurrent and contralateral symptoms, ultimately requiring a second surgical intervention (lumbar microdiscectomy). This case highlights the importance of early recognition of CES symptoms and the need for vigilant postoperative monitoring to detect recurrent or contralateral symptoms. Atypical presentations of CES should also be acknowledged to avoid delayed or missed diagnosis of this potentially devastating neurological condition.

## Introduction

Cauda equina syndrome (CES) is a neurosurgical emergency characterised by compression of the cauda equina nerve roots, leading to significant neurological impairment if not promptly treated. The term 'cauda equina' anatomically refers to the spinal nerves L2-L5, S1-S5, as well as the coccygeal nerve [1].

CES is a rare but critical spinal condition, affecting approximately one to three individuals per 100,000 in England, as mentioned in the National Getting it Right First Time (GIRFT) CES pathway [2]. Incidence of CES in the United Kingdom is 1.0-1.9 per 100,000 [3]. It often presents with radicular pain and/or lower back pain and demands prompt evaluation, diagnostic workup, and urgent intervention [4]. Failure to recognise CES or delays in surgical management can lead to irreversible complications, including paralysis of the lower limbs, bladder and bowel incontinence, and sexual dysfunction.

The most common cause of CES is lumbar disc herniation, particularly in the lower lumbar region. Other less frequent causes include spinal stenosis, trauma, malignancy, epidural abscess, and epidural haematoma. CES usually results from compression of the cauda equina by a substantial extrusion of disc material, often occurring in the context of degenerative or congenital spinal stenosis. Among disc herniations, central and posterolateral types are the most symptomatic, typically arising from axial biomechanical stresses on the lumbar discs. Standard CES diagnosis requires urgent clinical assessment for red flag symptoms, emergency MRI confirmation of spinal compression, and immediate surgical decompression within the critical 48-hour window, followed by structured postoperative rehabilitation. Despite being a well-recognised clinical entity, CES can present with varied and evolving symptoms, making early diagnosis particularly challenging [5]. 

Recurrent CES after an adequately performed decompression is uncommon, making this case significant for demonstrating persistent neural compromise despite adequate initial surgery. The patient's atypical contralateral symptoms and progression to second disc extrusion requiring repeat decompression highlight the importance of close clinical surveillance and a low threshold for MRI, for early detection and timely re-intervention when new or progressive neurological deficits develop after decompression.

## Case presentation

A 35-year-old healthy man presented to the emergency department (ED) with progressive lower back pain for three weeks radiating down the right leg, accompanied by worsening paraesthesia in the right foot, not associated with any weakness. He denied recent trauma and heavy lifting. His past medical history included chronic sciatica. 

Three weeks before presentation, the patient was seen by his general practitioner (GP) for new-onset pain radiating from the right hip down to the foot. At that time, he denied back pain, nocturnal pain, urinary or faecal incontinence, and altered perianal sensation. He remained fully mobile. On clinical examination, he appeared well, with normal gait and balance, and was found to have mild lumbar spinal tenderness on palpation. Neurological examination revealed normal lower limb muscle strength across all major muscle groups, including hip flexors/extensors, knee flexors/extensors, ankle dorsiflexors/plantar flexors, and toe movements. Sensory assessment demonstrated reduced sharp sensation over the right lateral foot. Deep tendon reflexes were preserved, and plantar reflexes were downgoing bilaterally, indicating normal upper motor neuron function. A clinical diagnosis of right-sided sciatica was made by the GP. The patient was started on amitriptyline, advised to continue nonsteroidal anti-inflammatory drugs (NSAIDs), and referred for a lumbar spine X-ray. He was educated about red flag symptoms and asked to urgently come to the ED if symptoms worsen. 

Upon arrival at the hospital, vital signs revealed a Glasgow Coma Scale (GCS) of 15/15, temperature of 37.1℃, heart rate of 87 beats/minute, blood pressure of 104/64 mmHg, and oxygen saturation (SpO₂) of 98% on room air. Routine blood tests showed no abnormalities of clinical concern. Neurological examination revealed no spinal tenderness, with intact sensation and motor function in all limbs. Digital rectal examination showed normal anal sphincter tone, no saddle anaesthesia, and no perianal numbness. Observations were within normal limits, and the bladder scan showed no urinary retention. He was treated with 100 mg rectal Diclofenac in the ED, which relieved the pain. A working diagnosis of lower back pain with right-sided sciatica was made, and he was discharged with analgesics, lansoprazole for gastric protection, and was instructed to return urgently if he developed leg weakness, urinary or faecal incontinence, and altered perianal sensation. 

The patient re-presented to the ED the next morning with worsening severe lower back pain (pain score 10/10), predominantly in the right lower back. The pain intensified with weight bearing and was relieved by lying flat, significantly limiting mobility. He denied urinary incontinence, limb weakness, saddle anaesthesia, fever, recent trauma, or heavy lifting. On examination, he appeared pale and clinically unwell. His vitals were within normal range on arrival. Neurological examination was unremarkable. Musculoskeletal assessment revealed tenderness over the posterior right hip, a weak straight leg raise on the right, and a positive right log roll test, leading to a working diagnosis of right sacroiliitis. In the ED, he was treated with IV paracetamol (1 g), oral morphine (10 mg), oral diazepam (5 mg), rectal Diclofenac (100 mg), and 1 L of IV normal saline over two hours. Again, routine blood tests showed no abnormalities of clinical concern. His pain improved, though right leg weakness and difficulty walking persisted. The orthopaedic and physiotherapy teams were involved at this point, and the patient was mobilised with crutches and discharged with safety netting advice, as the presentation did not raise suspicion of any signs of CES. He was also discharged with ongoing analgesia and diazepam, and a recommendation for GP follow-up to review and optimise his pain management. 

He then re-presented for the third time the following day with worsening pain despite regular analgesia, now describing it as “numbing” and “spasm-like.” He denied saddle anaesthesia, had no urinary voiding difficulties, and reported no bowel concerns. On examination, his GCS was 15/15, though he appeared diaphoretic and clammy. Observations revealed tachycardia (105 bpm) and elevated blood pressure (163/108 mmHg), with respiratory rate, temperature, and oxygen saturation within normal limits. Neurological examination showed full (5/5) power and intact sensation in all limbs. His lower limbs were cold to the touch, but pedal pulses were palpable bilaterally. Musculoskeletal examination revealed tenderness over the right hip, a full range of motion, and a straight leg raise test on the left, with a limited straight leg raise to 45 degrees on the right. A pelvic X-ray was performed, revealing no bony injury and preserved hip joint spaces, with only minimal degenerative changes and marginal osteophyte formation.

The case was discussed with the Orthopaedic Registrar, who attributed the presentation to an acute exacerbation or right-sided sciatica due to the lack of red flag CES symptoms. Gabapentin 300 mg was prescribed with gradual dose escalation over three days, and the GP was advised to review the patient after three days to determine whether to continue or taper treatment. He remained under outpatient physiotherapy care, with reassurance that any concerns would be escalated through their local multidisciplinary team. The patient was advised to return urgently if he developed new symptoms such as incontinence, saddle anaesthesia, or bilateral leg pain. 

The patient re-presented for the fourth time the following day with new-onset difficulty opening his bowels and reduced perianal sensation when wiping, but denied urinary incontinence or retention. He reported worsening lower back pain, radiating bilaterally down the posterior aspect of both legs to the feet, now more pronounced on the right. Despite ongoing analgesia, he rated the pain as 6/10. He continued to require crutches for mobility, with pain worsening when sitting upright and lying flat. 

**Figure 1:**
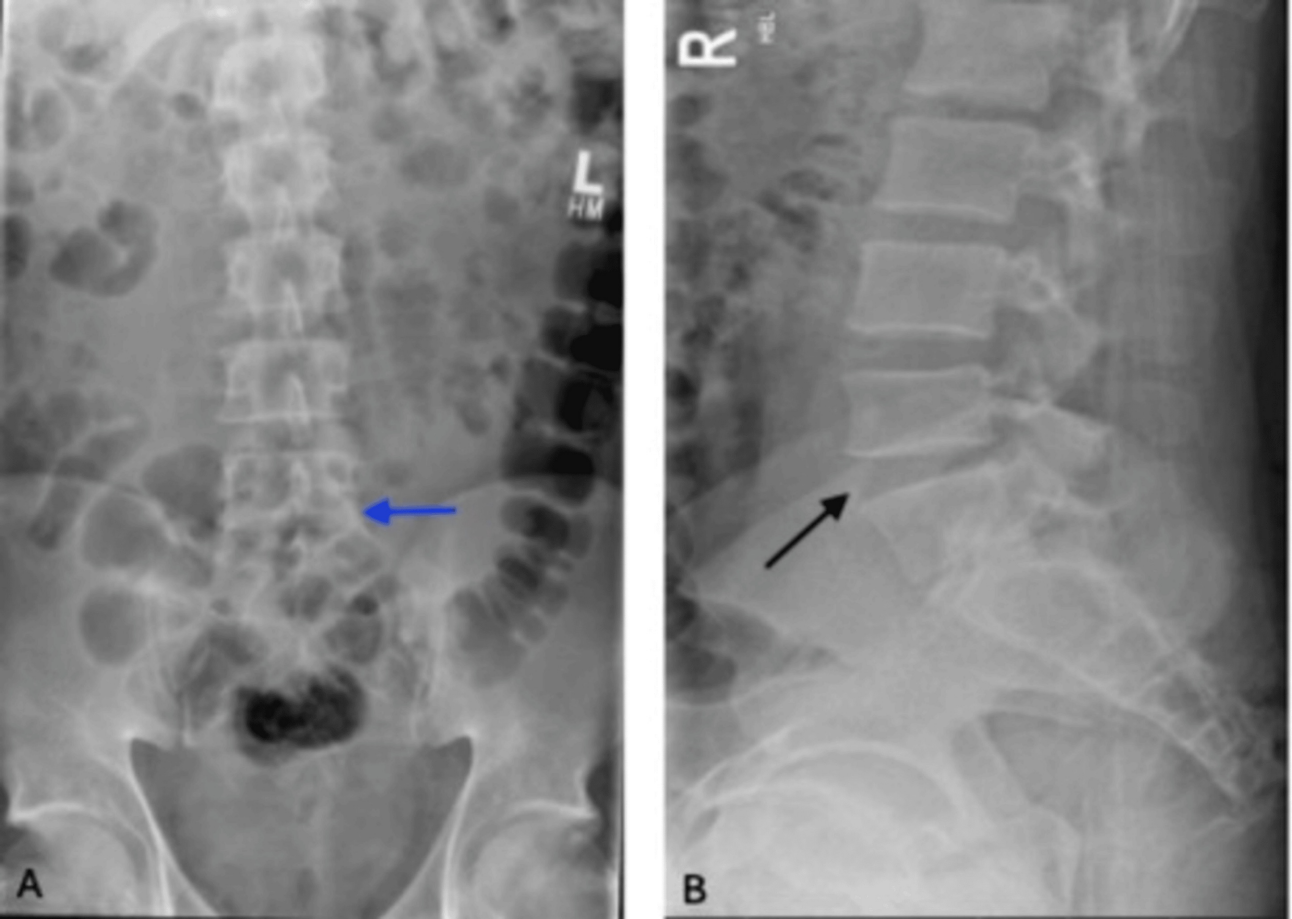
Plain X-rays of the lumbar spine (October 17, 2024). (A) Anteroposterior view showing degenerative changes in the lumbar spine with reduced intervertebral disc space at L5-S1 (blue arrow). (B) Lateral view demonstrating loss of lumbar lordosis with evidence of significant L5-S1 disc space narrowing and facet joint arthropathy (black arrow), consistent with underlying degenerative disc disease.

On assessment, his GCS was 15/15. Observations revealed elevated blood pressure (182/108 mmHg), with heart rate, respiratory rate, temperature, and oxygen saturation within normal limits. ECG showed a normal sinus rhythm. Examination revealed tenderness over the lower lumbar spine. Straight leg raise was limited to 30 degrees on the right and 60 degrees on the left. He had a positive right crossed straight leg raise test and bilateral positive sciatica stretch, which was worse on the right. Digital rectal examination showed reduced perianal sensation with preserved anal tone. Power and sensation in the lower limbs remained intact. A lumbar spine X-ray (Figure 1) demonstrated degenerative changes, reduced L5-S1 disc space, and facet joint arthropathy with loss of lumbar lordosis, consistent with degenerative disc disease. A bladder scan revealed a pre-void volume of 418 mL (normal < 400-500 mL) and a post-void residual of 23 mL. 

**Figure 2:**
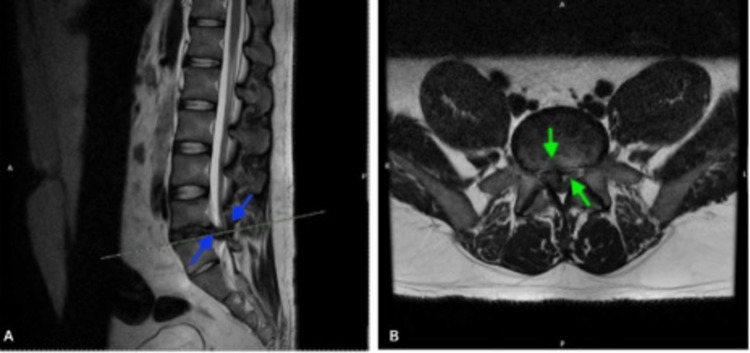
MRI lumbar spine (October 17, 2024). (A) Sagittal T2-weighted view showing a large central disc herniation at L4/L5 causing severe spinal canal stenosis and compression of the thecal sac (blue arrows). (B) Axial view demonstrating effacement of the CSF around the cauda equina nerve roots due to the disc herniation (green arrows), consistent with cauda equina syndrome.

The patient was then referred to orthopaedics, where he was assessed an urgent lumbar-sacral MRI scan was done, which revealed significant L4-L5 intervertebral disc herniation with right paracentral retropulsion, impinging on the cauda equina and causing right lateral recess stenosis and nerve impingement (Figure 2). An immediate referral was made to an external neurosurgery service, where he was accepted for urgent transfer for emergency neurosurgical intervention. 

Upon arrival, he consented to an L4 right-sided laminectomy with L4/L5 discectomy. The procedure and recovery were uncomplicated. He was discharged after three days with a follow-up appointment with the neurosurgical team in six weeks and a prescription for paracetamol, ibuprofen, and lansoprazole. 

Postoperatively, his urine output was monitored, and he did not experience further episodes of saddle anaesthesia. No additional imaging was required. He was planned for a structured three-phase rehabilitation program without restrictions, which included Initial mobilisation (0-2 weeks) with low-intensity exercises, progressive strengthening (2-6 weeks) with bridging and step-up exercises, and advanced rehabilitation (6-12 weeks) with weighted exercises to optimise functional recovery.

Less than three weeks after his procedure, when he had just finished week 2 of physiotherapy with low-intensity exercise and a few days of progressive strengthening, the patient re-presented to the ED with symptoms similar to those before surgery, but now on the opposite side and of milder intensity. He reported a three-day history of lower back pain radiating to the left leg, occasional numbness in the left thigh, and mild pain and stiffness at the surgery site. He denied any trauma, saddle anaesthesia, urinary incontinence, or bowel issues. Routine blood tests showed no abnormalities of clinical concern.

On examination, he had difficulty sitting upright, so the assessment was conducted supine. He was able to raise both legs to 45 degrees without pain. No spinal tenderness was noted, and his wound dressing was clean and dry. Digital rectal examination revealed reduced perianal sensation with normal anal tone. Neurological examination showed full power and sensation in both lower limbs, with a pre-void bladder volume of 109 mL and a post-void residual of 7 mL. 

**Figure 3:**
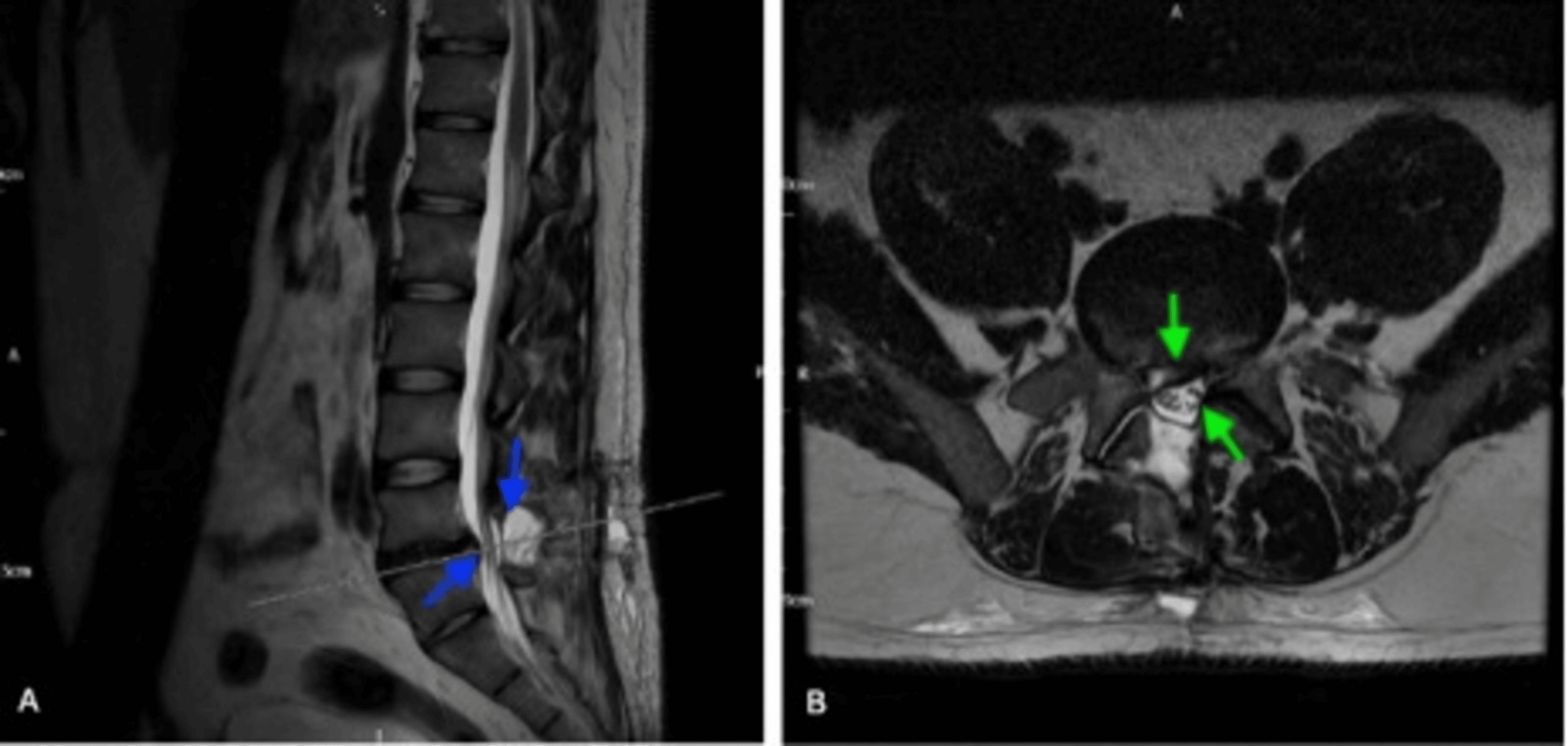
Postoperative MRI lumbar spine (November 5, 2024). Sagittal (A) and Axial (B) T2-weighted images showing postoperative changes at the L4/L5 level following surgical decompression for cauda equina syndrome. There is evidence of adequate decompression with resolution of central canal stenosis (blue arrows) and re-expansion of the thecal sac (green arrows).

Orthopaedics referred him for an urgent repeat lumbar-sacral MRI (Figure 3), which showed post-decompression changes at L4-L5 with a small seroma but no compression. A residual diffuse disc bulge at L4-L5 caused moderate narrowing of the left lateral recess and impinged on the left traversing L5 nerve root. A right-sided disc protrusion displaced the right traversing L5 nerve root. The specialist centre reviewed the MRI and recommended conservative management, advising no further surgery. He was educated about red flag warning signs and discharged home with follow-up instructions. 

**Figure 4:**
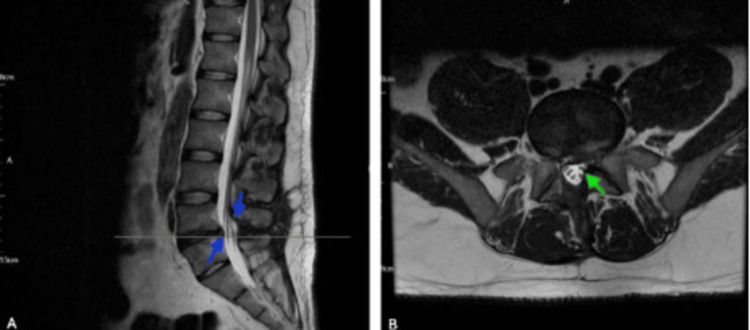
Follow-up postoperative MRI lumbar spine (February 22, 2025). (A) Sagittal T2-weighted image showing postoperative changes at the L4/L5 level. The blue arrows highlight the adequately decompressed thecal sac with no evidence of recurrent disc herniation or significant spinal canal stenosis. (B) Axial T2-weighted image demonstrating preserved central canal patency and no impingement of exiting or traversing nerve roots. The green arrow indicates mild postoperative epidural fibrosis, consistent with normal healing after lumbar decompression.

Four months after L4/5 decompression surgery, the patient presented with worsening lower back pain, left sciatica, and a single episode of faecal incontinence. He had been reviewed by the neurosurgical team a week prior and was educated on red flag symptoms. Examination showed preserved motor power and sensation, with mildly reduced perianal sensation. Neurosurgery advised an MRI, which revealed postoperative changes at L4/5 with no significant nerve root impingement (Figure 4).

Given the absence of new surgical indications, the patient was admitted for symptom management and discharged with safety-netting advice. He was referred to a spinal rehabilitation class and hydrotherapy due to impaired sensation in the L4/L5 distribution, along with pain, muscle guarding, and a limited range of movement, resulting in a lateral shift. 

He continued to frequently visit the ED (three times over 3.5 months) due to persistent tingling, reduced sensation, and weakness in his right leg. He was advised by the neurosurgical team that he may have ongoing sensory deficits that may never improve. A repeat lumbosacral MRI showed a recurrent right subarticular disc extrusion at L4-L5 with moderate spinal canal compromise and impingement of the right L5 nerve root. Mild crowding of the cauda equina was also noted, but CSF pockets were maintained, indicating preservation of cerebrospinal fluid spaces around neural structures despite the recurrent pathology. He was counselled regarding red flag symptoms and advised to continue hydrotherapy and regular analgesics.

A month after the latest ED visit, he re-presented to the ED with a new onset, one-day history of worsening saddle anaesthesia, bilateral hip pain, and ‘tingling’ down both legs. He denied any bowel or urinary incontinence. He was clinically stable, and routine bloods were unremarkable. On neurological examination, his power was 4/5 in his right leg, with reduced sensation over the right L4-5 dermatome. Mild tenderness was noted over his L3-L5 vertebrae. Reflexes were normal bilaterally. Pre and post-bladder scan showed no retention. 

**Figure 5:**
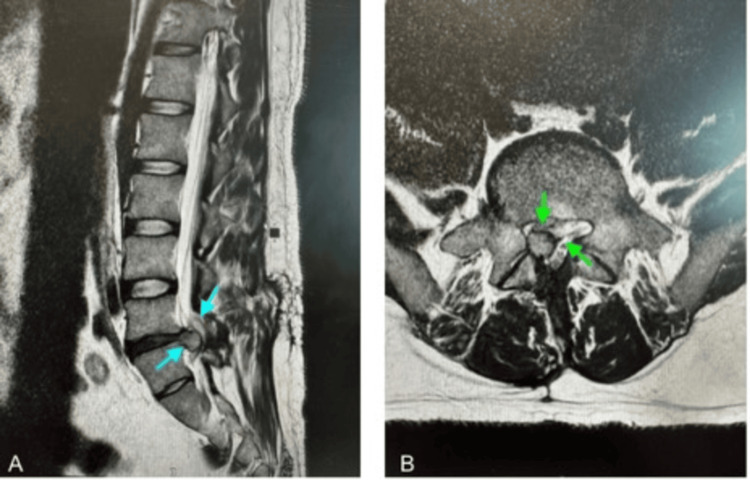
T2-weighted MRI images of the lumbar spine demonstrating cauda equina compression (June 7, 2025). (A) Sagittal T2-weighted MRI shows a significant disc protrusion at the L4-L5 level (blue arrows), with loss of normal CSF signal around the cauda equina, consistent with compression. Thecal sac effacement is noted with obliteration of the anterior epidural fat. The conus medullaris terminates at the normal level (approximately L1), above the site of compression. (B) Axial T2-weighted MRI at the L4-L5 level shows a large central disc herniation with severe narrowing of the spinal canal (green arrows). The herniated disc material is compressing the cauda equina nerve roots bilaterally, with obliteration of the lateral recesses and compromise of the traversing nerve roots.

His case was discussed with orthopaedics and the neurosurgical centre. A repeat MRI showed interval worsening of L4-L5 disc extrusion, now causing moderate to severe canal stenosis and right L5 nerve root impingement (Figure 5).

The neurosurgical centre advised that the clinical picture was not consistent with typical CES, but that, given the enlargement of the disc fragment and the progression of his symptoms, he would need an urgent neurosurgical assessment. He was then transferred to the neurosurgical centre for urgent assessment and management. Following specialist assessment, he had a lumbar microdiscectomy at the L4/L5 level. His recovery was uneventful. He was discharged with crutches after a rehabilitation assessment, advised to continue analgesia, provided with safety-netting advice, and scheduled for ongoing follow-up assessments. 

He was reviewed by the rehab team a week later, as part of his ongoing review. They observed him walking with a normal gait but with stiff and guarded movements. He was instructed to avoid excessive flexion, extension, or rotation of his lumbar spine, avoid handling heavy loads, and use the log-roll technique when getting in and out of bed. He was encouraged to do gentle exercises and continue hydrotherapy to enhance recovery from sensory deficits.

**Table 1:** Summary of the patient’s symptoms, examination findings, imaging, and interventions over time, illustrating the clinical progression and management decisions.

Timeline	Presentation	Imaging	Intervention
GP visit	New-onset right hip-to-foot radiating pain No red flags (saddle anesthesia/ urinary incontinence)	None	Diagnosed with right-sided sciatica; referred for lumbar spine X-ray; advised on red flag symptoms
Day 1 (1^st ^ED visit; 3 weeks after GP Visit)	Progressive lower back pain radiating down the right leg; worsening paraesthesia in the right foot.	Bladder scan- No urinary retention	Discharged with analgesia, and advised on red flags symptoms
Day 2 (2^nd^ ED visit)	Worsening right-sided lower back pain (10/10); limited mobility;No red flags (saddle anesthesia/ urinary incontinence)	None	Pain management ; diagnosed with right sacroiliitis. Mobilised with crutches; discharged with analgesia with GP follow-up
Day 3 (3^rd ^ED visit)	Worsening pain described as "numbing" and "spasm-like”; no saddle anaesthesia, urinary/bowel issues; sweaty, clammy, tachycardic (105 bpm), hypertensive (163/108 mmHg)	Pelvic X-ray: no bony injury and preserved hip joint spaces, with only minimal degenerative changes and marginal osteophyte formation.	Attributed to acute exacerbation of chronic sciatica; continue analgesics ; discharged with outpatient physiotherapy; advised on red flag symptoms
Day 4 (4^th^ ED visit)< 24 hours after pelvic X-ray	Worsening lower back pain radiating bilaterally to feet (worse on right); difficulty opening bowels; reduced perianal sensation; no urinary issues; hypertensive (182/108 mmHg)	Lumbar spine X-rays (Figure 1, October 17, 2024): degenerative changes noted,reduced L5-S1 disc space and facet joint arthropathy with loss of lumbar lordosis, consistent with degenerative disc disease. Urgent LS MRI (Figure 2, October 17, 2024): L4-L5 disc herniation compressing cauda equina	Urgent transfer to neurosurgery - emergency L4 right-sided laminectomy with L4/L5 discectomy
Day 7 (Postoperative discharge)	Initial postoperative improvement; engaged with physiotherapy; nil acute postoperative complications.	None	Discharged after three days with analgesics with neurosurgical follow-up in six weeks
Day 23	Contralateral (left-sided); lower back pain radiating to left leg; occasional numbness in left thigh; mild pain/stiffness at surgery site; no trauma, saddle anaesthesia, or incontinence.	Urgent LS MRI (Figure 3, November 5, 2024): post-decompression changes, residual disc bulge impinging left L5 nerve root, no significant compression.	Conservative management recommended by neurosurgeons; discharged with safety netting (education on red flags symptoms).
Day 132 (~4.5 months)	Worsening lower back pain; left sciatica; single episode of faecal incontinence; preserved motor power/sensation; mildly reduced perianal sensation	LS MRI (Figure 4, February 22, 2025): postoperative changes, no recurrent herniation or stenosis, mild epidural fibrosis	Admitted for symptomatic management and referred to spinal rehabilitation and hydrotherapy.
Frequent ED visits (three ED visits in ~ 3 months)	Persistent tingling, reduced sensation, and weakness in right leg; advised possible ongoing sensory deficits	Repeat LS MRI: recurrent right subarticular disc extrusion at L4-L5, moderate spinal canal compromise, right L5 impingement, mild cauda equina crowding	Patient education regarding red flag symptoms ; encouraged hydrotherapy and analgesics; no surgical intervention advised by neurosurgical team.
Day 237 (~8 months) 1 month after previous repeat MRI	New-onset worsening saddle anaesthesia; bilateral hip pain; tingling down both legs; no urinary/bowel incontinence; power 4/5 right leg; reduced sensation right L4-5 dermatome	Repeat LS MRI (Figure 5, June 7, 2025): worsening L4-L5 disc extrusion, moderate to severe canal stenosis, cauda equina compression	Patient transferred for urgent neurosurgical assessment; lumbar microdiscectomy at L4/L5 level performed.
Day 240 (Post second surgery discharge)	Uneventful recovery	None	Patient education regarding red flag symptoms; discharged with crutches after rehab assessment; analgesia; ongoing follow-up
Day 244 (1 week post second surgery)	Walking with normal gait but complaining of leg stiffness and guarded movements	None	Rehab review; use log-roll technique; gentle exercises; continue hydrotherapy.

## Discussion

This case illustrates the diagnostic and management challenges associated with CES, a rare but critical neurological complication of LDH. CES occurs in approximately 1-3% of patients with LDH, a prevalence consistent across both international and United Kingdom data [4]. In this case, the patient's initial presentation with chronic sciatica and ongoing lower back pain likely masked the evolution of progressive neurological deficits, contributing to a delayed diagnosis. This highlights the need for a high index of suspicion in patients presenting with persistent or evolving back pain, particularly in younger individuals or those with emerging perianal sensory changes.

Diagnosing CES is often difficult due to the variability and ambiguity of early symptoms. Key clinical features such as back pain, bladder dysfunction, saddle anaesthesia, and lower limb weakness may not occur simultaneously. Instead, they may develop acutely or progress insidiously over days to weeks, as seen in this patient with recurrent and gradually worsening symptoms [5].

LDH, the underlying pathology in this case, occurs when disc material (nucleus pulposus or annulus fibrosus) extends beyond the normal disc margins and compresses adjacent spinal nerve roots. LDH is a common cause of sciatica and lower back pain, frequently requiring surgical intervention [6]. Risk factors associated with disc herniation include being overweight, male sex, age over 40 years, a history of back problems, and engaging in physically demanding work or leisure activities involving heavy lifting [7].

Surgical decompression remains the cornerstone of CES treatment. However, even when performed promptly, functional recovery may be incomplete. Approximately 20-30% of patients with CES continue to experience residual bladder, bowel, or sexual dysfunction despite prompt surgery [8]. This suggests that irreversible nerve injury may already be present at the time of intervention. In this case, right-sided L4 laminectomy and L4/L5 discectomy initially led to symptomatic relief. However, the patient subsequently developed recurrent radicular symptoms and perianal paraesthesia, a recognised complication of lumbar surgery. Recurrent symptoms after decompression can result from residual or recurrent disc herniation, postoperative seroma formation, or epidural fibrosis, as demonstrated on this patient’s serial MRIs [9,10]. Subsequent lumbar microdiscectomy was ultimately required, emphasising the need for ongoing clinical reassessment and timely imaging in patients with evolving symptoms.

The patient's multiple emergency presentations, despite appropriate initial conservative management and red flag education, reflect the fluctuating and progressive nature of CES. Early symptoms such as unilateral sciatica, paraesthesia, or non-specific lower back pain often mimic benign radiculopathy, making early differentiation difficult and increasing the risk of delayed diagnosis. Repeated neurological assessment, patient education regarding red flag symptoms, accurate documentation of symptom progression, and early access to MRI are critical in evaluating patients with unresolved or atypical presentations of lower back pain [11]. In this case, serial imaging and collaboration across emergency, orthopaedic, neurosurgical, and physiotherapy teams enabled appropriate escalation and intervention.

Despite adequate anatomical decompression, the patient continued to experience neuropathic symptoms and functional limitations. Long-term studies show that 20-30% of patients with CES may report persistent deficits in motor or sensory function, bladder control, or sexual function, even after surgery [12]. Postoperative epidural fibrosis is increasingly recognised as contributing to chronic neuropathic symptoms following CES decompression, causing persistent pain through neural microvasculature disruption and nerve root tethering in up to 30% of lumbar laminectomy cases. Preclinical studies show interventions like polypropylene mesh can reduce fibrous scarring by limiting fibroblast proliferation. The mild epidural fibrosis observed on follow up MRI (Figure 4) likely contributes to this patient's persistent sensory deficits, emphasising the need for preventive strategies [13].

Postoperative complications after lumbar decompression includes surgical site infections and urinary tract infections; in a study of 524 patients, 8% required re-operation and 17% were readmitted within 30 days, underscoring the importance of close postoperative monitoring [14]. These outcomes underline the importance of timely diagnosis, but also reinforce that postoperative rehabilitation, physiotherapy, and patient education are vital components of long-term care .

When the diagnosis of CES is delayed or neurological recovery is incomplete, this can lead to significant medical, legal and psychosocial consequences. As seen in this case, multidisciplinary coordination, timely imaging, and prompt surgical intervention followed by structured rehabilitation, are essential for optimising outcomes and improving quality of life.

## Conclusions

CES necessitates prompt recognition and urgent intervention to minimise the risk of irreversible neurological damage. This case highlights the diagnostic challenges of CES with an insidious onset, atypical progression, and fluctuating symptoms, which initially mimicked benign sciatica. It underscores the critical importance of maintaining a high index of suspicion in patients with evolving or recurrent neurological symptoms, even in the absence of classic red flags. Optimal outcomes require a multidisciplinary approach, integrating neurosurgical, radiological, and rehabilitative expertise. 

The patient’s postoperative course further highlights the need for vigilant, long term follow-up to detect residual or recurrent disc herniation and prevent secondary neurological deterioration. Managing chronic CES symptoms remains challenging, as surgical decompression may resolve compression but cannot fully reverse established neurological deficits and long-term sequelae. Optimal outcomes in CES require a coordinated multidisciplinary approach, involving neurosurgical, radiological, and rehabilitative teams, along with regular neurological assessments for early identification of complications.
